# Impact of LINE-1 hypomethylation on the clinicopathological and molecular features of colorectal cancer patients

**DOI:** 10.1371/journal.pone.0197681

**Published:** 2018-05-24

**Authors:** Tai-Chuan Kuan, Pei-Ching Lin, Shung-Haur Yang, Chun-Chi Lin, Yuan-Tzu Lan, Hung-Hsin Lin, Wen-Yi Liang, Wei-Shone Chen, Jen-Kou Lin, Jeng-Kai Jiang, Shih-Ching Chang

**Affiliations:** 1 Division of Colon & Rectal Surgery, Department of Surgery, Taipei Veterans General Hospital, Taipei, Taiwan; 2 Department of Surgery, Faculty of Medicine, School of Medicine, National Yang-Ming University,Taipei, Taiwan; 3 Department of Clinical Pathology, Yang-Ming Branch, Taipei City Hospital, Taipei, Taiwan; 4 Department of Health and Welfare, University of Taipei, Taipei, Taiwan; 5 Department of Pathology, Taipei Veterans General Hospital, Taipei, Taiwan; National Cancer Center, JAPAN

## Abstract

Recent studies suggest that aberrant DNA methylation might occur early and commonly in colorectal tumorigenesis. In 111 normal subjects, the mean LINE-1 methylation level of peripheral blood was 81.0 ± 5.7%. Of 143 colorectal cancer (CRC) patients, the mean level of LINE-1 methylation was 60.5 ± 12.5%. We defined below 60% as cut-off value of LINE-1 hypomethylation, and 93 cases (65.0%) had LINE-1 hypomethylation in the tumor tissue. LINE-1 hypomethylation was not associated with any other clinical features. There was a trend that LINE-1 hypomethylation tumors were associated with advanced disease, but it did not reach statistical significance. There was no significant association between mutations of 12 genes, MSI-high, EMAST, and LINE-1 hypomethylation level. The median follow-up was 61.2 months. Five-year disease-free survival (DFS) and overall survival curves of patients with LINE-1 hypomethylation tumors were significantly lower than those of patients with normal LINE-1 methylation tumors (p = 0.032 and 0.001, respectively). Multivariate analysis showed that only TNM staging was an independent prognostic factor for CRC patients including DFS and overall survival (OS). LINE-1 did not impact patients’ outcomes in multivariate analysis including DFS and OS. In conclusion, LINE-1 hypomethylation is marginally related to advanced stage CRC and impacts patients’ outcomes in univariate analysis.

## Introduction

Colorectal cancer (CRC) has become the most common cancer in Taiwan. More than 15,000 new diagnosed CRC cases were reported each year since 2013 [1]. As consistent with other models describing colorectal cancer originating from progressive accumulation of genetic and epigenetic alterations [2–4], the molecular analysis in our previous studies[5–7] showed that CRCs had higher frequency of mutations in APC, TP53, and KRAS. These genomic alterations associating with chromosomal instability or aneuploidy were found in the majority of CRC cases [3,4,7]. In the screening of Lynch syndrome, analysis of microsatellite instability(MSI) and immunohistochemistry of mismatch-repair proteins showed that 10–15% CRC cases were MSI-high or had deficient MMR proteins.[8–11] Overall 2–3% CRC cases were found to have germline mutations in the mismatch repair system.(8,9,12) Hypermethylation of the MLH1 gene promoter resulting in silence of MMR proteins is another cause of MSI-high [12–14].

Recently, whole-genome methylation analyses of CRCs, precursor lesions, and normal colorectal mucosa provided evidence that aberrant DNA methylation might occur early in colorectal tumorigenesis and is a common event in CRC [15,16]. DNA methylation is known to add a methyl group to the fifth carbon atom of a cytosine ring at the “CG” dinucleotide sequence. Global DNA methylation occurs within highly repetitive DNA sequences, such as long interspersed nucleotide elements (LINE-1) and short repetitive sequences such as Alu repeats [17–18].

LINE-1 are retrotransposon elements composing about 17–18% of the human genome [19]. Line-1 might inactivate gene function through insertional mutagenesis or aberrant splicing or LINE-1-mediated insertions could result in disease through target-site deletions [20]. In normal tissue, LINE-1 is highly methylated and inactivated, but some of them still retaining the capacity to retrotranspose themselves to new genomic locations[21]. In cancer tissue, LINE-1 methylation is decreased [19–21]. Hypomethylation of Line-1 has been considered to be associated with increased retrotransposon activity[22], induced genomic instability and result in further progress of cancer formation [23–27]. Because Line-1 is highly abundant and randomly distributed throughout the genome, LINE-1 methylation could be used as a surrogate marker of global DNA methylation and is confirmed in several studies [18,23–30].

In this study, LINE-1 methylation levels in the peripheral blood were analyzed to understand the distribution of LINE-1 methylation level. In colorectal cancer tissue, cut-off value of LINE-1 methylation was determined and correlated with clinicopathological features and molecular alterations, including gene mutations, MSI, and elevated microsatellite alterations at selected tetranucleotides (EMAST).

## Materials and methods

### Clinical data

One hundred eleven healthy individuals, with informed consent, were enrolled from volunteer blood donors who had no history of malignant disease. DNA of peripheral blood from normal individuals was extracted and stored in Taipei Veterans General Hospital Biobank. One hundred forty-three samples were selected randomly from a prospective collected database consisting of 1505 patients with colorectal cancer who received surgery at the Taipei Veterans General Hospital between 2000 and 2010 [5–7]. This database excluded patients died of surgical complications, rectal cancer patients receiving preoperative chemoradiotherapy, and patients receiving emergency operations because of cancer complications. We prospectively collected data including age, sex, personal and family medical history, location of tumor, TNM stage, and other pathological prognostic features and follow-up condition. Colon length between the cecum and rectosigmoid colon was defined as the colon. The rectum was within 15 cm of the anal verge. After operation, patients were informed to be monitored every three months in the first two years and semiannually thereafter. Every clinical visits, patients received examinations physical, digital rectal examination, carcinoembryonic antigen and CA-199 analysis, chest radiography, abdominal sonogram. The computerized tomography was arranged if any abnormal finding was found. Proton emission tomography or magnetic resonance imaging was arranged for patients with elevated levels of carcinoembryonic antigen but an uncertain site of tumor recurrence.

### Source of samples

After approval by the Institutional Review Board of Taipei Veterans General Hospital (number 2013-11-013CCF), DNA of peripheral blood and samples of tumors were obtained from the Biobank. Tumor DNA was extracted using a QIAamp DNA Tissue Kit (Qiagen, Valencia, CA, USA) according to the manufacturer’s recommendations. Quality and quantity of DNA were confirmed using a Nanodrop 1000 spectrophotometer (Thermo Scientific).

### MassArray-based mutation characterization

The identification of 139 mutations in 12 genes detected by the MassDetect CRC panel (v2.0) was extracted from our previous studies [5,6]. In brief, polymerase chain reaction (PCR) and extension primers for the mutations were designed using MassArray Assay Design 3.1 software (Sequenom, San Diego, CA, USA). PCR products from the multiplexed reactions were spotted onto SpectroCHIP II arrays, and DNA fragments were resolved on a MassArray Analyzer 4 System (Sequenom). Each spectrum was then analyzed using Typer 4.0 software (Sequenom) to identify mutations. We defined a 5% abnormal signal as a putative mutation. Putative mutations were then filtered by manual review. Our previous study had verified the concordance between MassArray and Sanger sequencing up to 99%.(6)

### MSI analysis

According to international criteria, five reference microsatellite markers were used to determine MSI: D5S345, D2S123, BAT25, BAT26, and D17S250. Primer sequences for these genes were obtained from GenBank (https://blast.ncbi.nlm.nih.gov/Blast.cgi). MSI detection was performed as previously described [7]. The specific microsatellite sequence was amplified with polymerase chain reactions(PCR). The PCR products were denatured and analyzed by 5% denaturing polyacrylamide gels using ABI-3730 analyzer(Applied Biosystems, CA,USA), and results were revealed using GnenScan analysis software(Applied Biosystems, CA,USA). The samples with ≥ 2 MSI markers were defined as having high MSI, and those with 0–1 MSI markers were classified as microsatellite stable.

### EMAST analysis

According to the definition by some studies [31,32], five tetranucleotide repeats markers were used to determine EMAST: D20S82, D20S85, D8S321, D9S242, and MYCL1. Primer sequences for these genes were obtained from GenBank (https://blast.ncbi.nlm.nih.gov/Blast.cgi). EMAST detection was performed as previously described [31,32] and the technical details were similar to those of MSI analysis. Samples with ≥ 2 tetranucleotide markers were defined as having EMAST, and those with 0–1 tetranucleotide markers were classified as without EMAST.

### Methylation-Specific PCR for LINE-1

The methylation status of LINE-1was examined using the EpiTect Methyl II PCR Array [33]. Briefly, input genomic DNA was aliquoted into four equal portions and subjected to mock, methylation-sensitive, methylation-dependent, and double restriction endonuclease digestion. After digestion, the enzymatic reactions were mixed directly with the quantitative PCR (QPCR) master mix and were dispensed into a PCR array plate containing pre-aliquoted primer mixes. The sequences of the primers used for methylation-specific PCR were 185F: 5’-CATTGCCTCACCTGGGAAGC-3’ and 431R:5’-CAGCCTCGTTGCCGCCTTG-3’. The assay amplified a region of LINE-1 element (position 305 to 331 in accession No. X58075) including 4 CpG sites.

Real-time PCR was conducted using the specified cycling conditions. Finally, the raw change in the threshold cycle number (ΔCt) was pasted into a data analysis spreadsheet, which automatically calculated the relative quantities of methylated and unmethylated DNA. The average of the relative amounts of C in the 4 CpG sites was used as overall LINE-1 methylation level in a given sample.

### Statistical analysis

The statistical endpoint for disease-free survival(DFS) was defined to have disease since the date of diagnosis or even surgery. The overall survival(OS) was measured from the date of surgery or diagnosis to the date of death from any cause. Patients not known to have died were censored on the date of their last follow-up. The survival curves were plotted using Kaplan-Meier method and compared using the log-rank test. Cox regression univariate and multivariate analyses were performed to determine the impact of clinicopathological features on DFS and OS. The relationship between the genotype frequency and clinicopathological features were analyzed using the chi-square test and 2-tailed Fisher’s exact procedure. Numerical values were compared using Student’s t-test. Data were expressed as mean ± standard deviation. Statistical significance was defined as p < 0.05. Statistical analyses software was SPSS for Windows (version 16.0).

### Results and discussion

**Fig 1:**
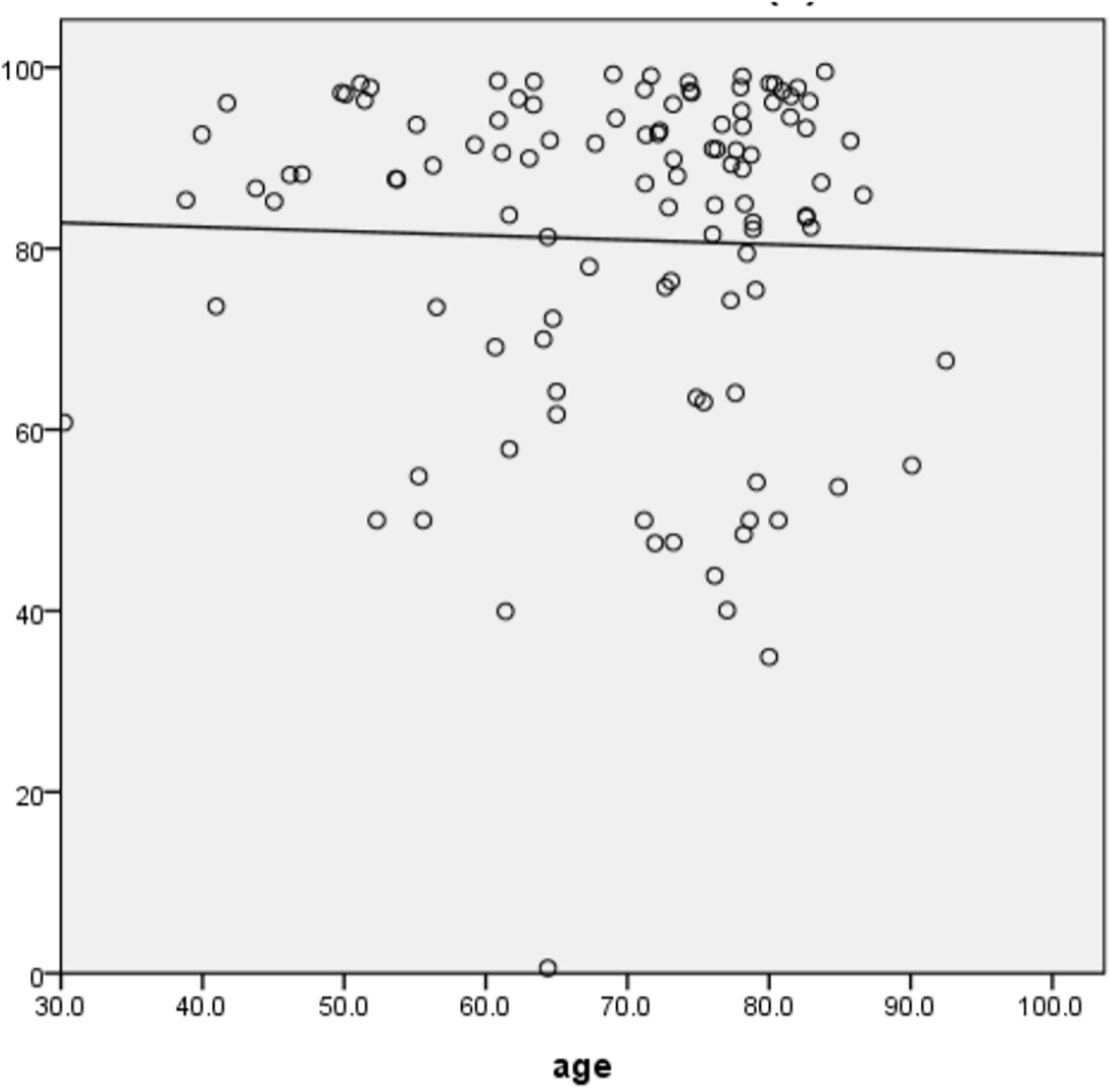
Distribution of LINE-1 methylation level in different age group. X axis: age. Y Axis: Line-1 methylation, expression with percentage.

In 111 normal subjects, LINE-1 methylation was analyzed in DNA of peripheral blood. As shown in Fig 1, the mean LINE-1 methylation level in the peripheral blood was 81.0 ± 5.7% (median 88%, range 35–100%). Of the normal subjects, 37.6% had < 80% methylation of LINE-1in the peripheral blood. CRC patients consisted of 93 men (65.0%) and 50 women (35.0%). The mean age at treatment was 79.6 ± 12.4 years (range: 38.8–85.2 years; median: 70.9 years). Ninety-eight (68.5%) and 45 (31.5%) cases were located in the colon and rectum, respectively. There were 33(23.1%) stage I,32 (22.4%) stage II, 34 (23.8%) stage III and 44 (30.8%) stage IV disease by tumor staging (TNM) respectively. Thirty-nine (27.3%), 10 (7.0%), and 11 (7.7%) tumors had lymphovascular invasion, mucinous component, and poor differentiation in histology, respectively. Twenty (14.0%) and 18 (12.6%) patients had cancers with MSI-high or EMAST, respectively.

Of the 143 CRC patients, the mean level of LINE-1 methylation was 60.5 ± 12.5% (median 65.2%, range 22.9–79.0%). According to previous studies [34,35], we defined below 60% as the threshold for LINE-1 hypomethylation, with 93 cases (65.0%) meeting this definition.

As shown in Table 1, tumors with the LINE-1 hypomethylation were not significantly associated with any other clinical features, including age, gender, and location. There was a trend that LINE-1 hypomethylation tumors associated with advanced disease, but it did not reach statistical significance (p = 0.093). The LINE-1 hypomethylation tumors had 21.5% and 36.6% stage III and stage IV disease, respectively. In contrast, the normal LINE-1 methylation tumors had 28% and 20% stage III and stage IV disease, respectively. The other pathological features, including lymphovascular invasion (LVI), mucinous histology, and poor differentiation were not significantly associated with LINE-1 hypomethylation tumors.

**Table 1:** Clinicopathological features of patients with hypo and normal methylation of Line-1 in colorectal cancer tissue.

	<60% methylation	> = 60% methylation	p
Age	70.76±12.7	68.5±12.3	0.542
Gender(female)	29(31.2)	21(42.0)	0.204
Location			
Colon	65(69.9)	33(66.0)	0.707
Rectum	28(62.2)	17(34.0)	
TNM stage			
I	17(18.3)	16(32.0)	0.093
II	22(23.7)	10(20.0)	
III	20(21.5)	14(28.0)	
IV	34(36.6)	10(20.0)	
Lymphovascular Invasion(+)	26(28.0)	13(26.0)	0.846
Lymphovascular Invasion(-)	67(72.0)	37(74.0)	
Differentiated poorly(+)	9(9.7)	2(4)	0.329
Differentiated poorly(-)	84(93.5)	48(96)	
Mucinous histology(+)	6(6.5)	4(8.0)	0.093
Mucinous Histology(-)	87(93.5)	46(92.0)	

Hypomethylation defined as <60% methylation

As shown in S1 Table, there was no significant association between mutations of 12 genes, MSI-high, EMAST, and tumor LINE-1 methylation level. Because of rarity in individual gene mutation, mutation in genes assuming to have function in similar pathway were organized together. However, we could not find any association between tumor LINE-1 methylation level and alterations of molecular pathways(Table 2).

**Table 2:** Molecular alterations between patients with hypo and normal methylation of Line-1 in colorectal cancer tissue.

Alterations in Pathway	<60% methylation		>60% methylation		p value
	Case no:93	(%)	Case no:50	(%)	
APC- TP53- FBXW7	52	55.9	33	66.0	0.286
KRAS- NRAS- HRAS	38	40.9	23	46.0	0.597
BRAF	10	10.8	5	10.0	1.000
PI3KCA-PTEN- AKT1	8	8.8	4	8.0	1.000
TGFbR- SMAD4	5	5.4	3	6.0	1.000
MSI-high	11	11.8	9	18.0	0.322
EMAST	13	14.0	5	10.0	0.603

Hypomethylation defined as <60% methylation

**Fig 2:**
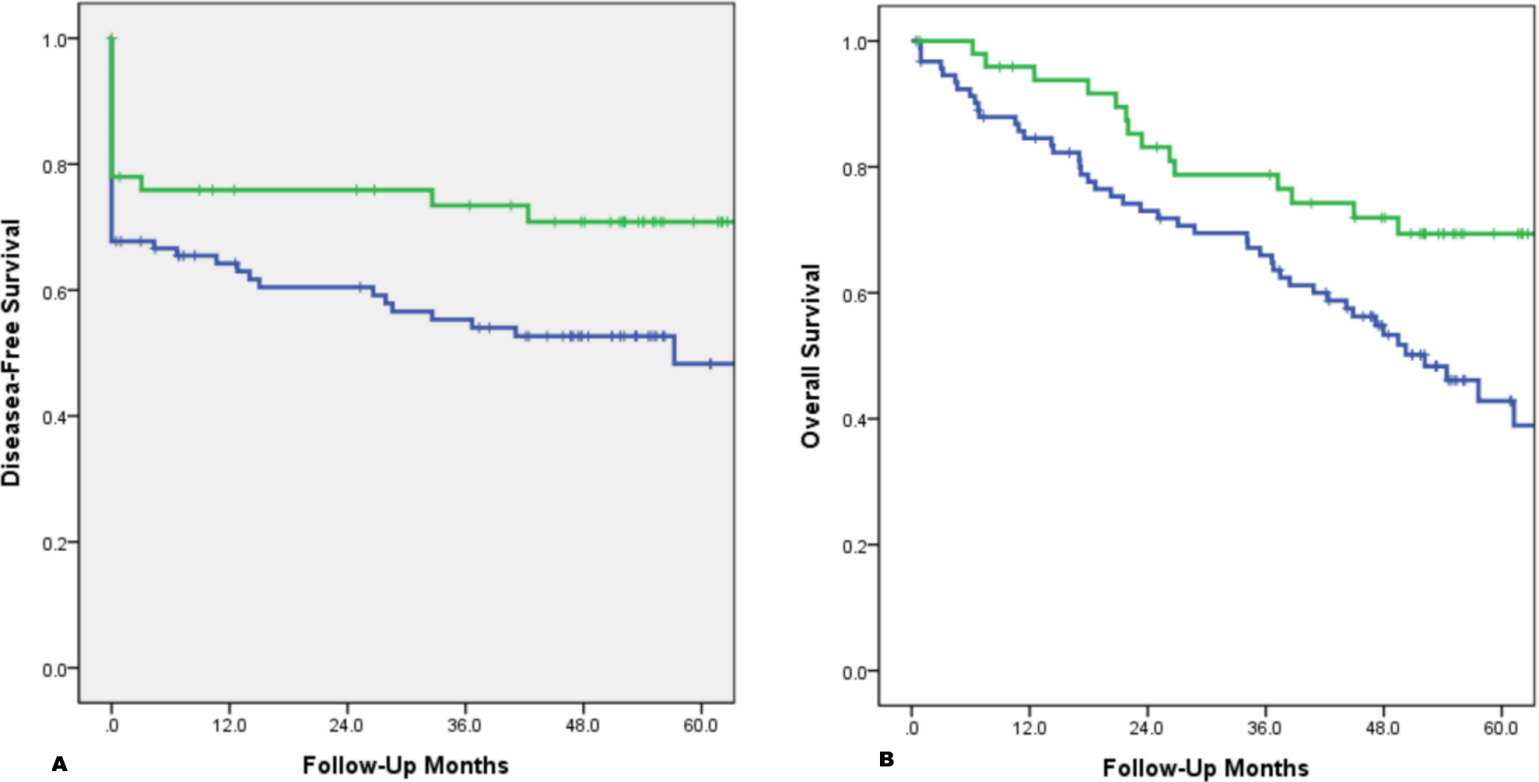
A) Green Line: 5-year disease-free survival of patients with normal LINE-1 methylation(76%) Blue Line: 5-year disease-free of patients with LINE-1 hypomethylation(41%, p = 0.001). B) Green Line: 5-year overall survival of patients with normal LINE-1 methylation(76%) Blue Line: 5-year overall survival of patients with LINE-1 hypomethylation(41%, p = 0.001).

The median follow-up was 61.2 months. There were 61 patients who developed metastatic disease, including liver (25), lung (17), peritoneal (12), and others (8). The five-year disease-free survival (DFS) curve of patients with LINE-1 hypomethylation tumors was 52%, significantly lower than that of normal LINE-1 methylation tumor patients (78%, p = 0.032; Fig 2A). In addition, the five-year overall survival (OS) curve of patients with LINE-1 hypomethylation tumors was 41%, significantly lower than that of normal LINE-1 methylation tumor patients (76%, p = 0.001; Fig 2B). The Cox regression model enrolling factors including TNM staging, LVI, mucinous histology, tumor differentiation and tumor LINE-1 hypomethylation (Tables 3 and 4) showed that only TNM staging was an independent prognostic factor for colorectal cancer patients including DFS (HR = 3.14, 95%; CI: 2.20–4.47) and OS (HR = 4.01, 95%; CI: 2.61–6.61).

**Table 3:** Univariate and multivariate analysis for disease-free survival.

	Univariate analysis			Multivariate analysis		
	HR	95% CI	p	HR	95% CI	p
TNM	3.29	2.36–4.57	<0.001	3.14	2.20–4.47	<0.001
Lymphovascular invasion	2.67	1.62–4.39	<0.001	1.99	1.18–3.38	0.010
Mucinous Histology	2.51	1.14–5.53	0.023	1.92	0.84–4.40	0.119
poor differentiation	1.03	0.37–2.77	0.99	1.21	0.43–3.43	0.707
Line-1 hypomethylation	1.86	1.07–3.25	0.027	1.50	0.87–2.75	0.132

HR: Hazard ratio; CI: confidence interval

**Table 4:** Univariate and multivariate analysis for overall survival.

	Univariate analysis			Multivariate analysis		
	HR	95% CI	p	HR	95% CI	p
TNM	4.26	2.79–6.48	<0.001	4.01	2.61–6.61	<0.001
Lymphovascular invasion	2.21	1.30–3.73	0.003	1.20	0.70–2.05	0.503
Mucinous Histology	1.82	0.78–4.26	0.165	2.10	0.51–8.19	0.307
poor differentiation	2.29	0.56–9.43	0.249	1.44	0.61–3.43	0.409
Line-1 hypomethylation	1.80	1.01–3.28	0.049	1.20	0.65–2.23	0.555

HR: Hazard ratio; CI: confidence interval

This study provided three major contributions. First, the cut-off value of tumor LINE-1 methylation could be defined at 60%. Second, LINE-1 hypomethylation was not associated with mutation of the genes studied including MSI-high, and EMAST. Third, LINE-1 hypomethylation was insignificantly associated with advanced disease. Further, hypomethylation of LINE-1 in tumor tissue impacted patients’ outcomes including OS and DFS in univariate analysis but not in multivariate analysis.

Our series showed that LINE-1 methylation in normal subjects was near 81% in average. As shown in previous studies, the average LINE-1 methylation of other normal tissues was approximately 70–90%, including kidney, colon, stomach, and peripheral blood [35–37]. In our series, 37.6% of normal subjects had LINE-1 methylation lower than 80% in the peripheral blood. Previous studies demonstrated that LINE-1 hypomethylation was usually related to genomic instability and resulted in some neoplasms [28,38–41]. These groups of cases with LINE-1 lower than 80% deserved to be closely monitored for future disease development.

The LINE-1 methylation level in our CRC tumors was approximately 60% (median 65.2%, range 22.9–79.0%), similar to the large-scale study. In the Nurses’ Health Study and the Health Professionals Follow-Up Study, in 1121 CRC patients, tumor LINE-1 methylation level ranged from 23.1% to 93.1% with a mean of 62.7 ± 9.4% [42]. In another study of 217 CRC patients, tumor LINE-1 methylation level ranged from 24 to 68% with a mean of 54.3 ± 7.5% [13]. Therefore, the definition of less than 60% as the cut-off value of LINE-1 hypomethylation was reasonable.

According to this definition, 65% of cases had LINE-1 hypomethylation. In contrast to previous studies showing that LINE-1 hypomethylation was associated with higher pN stage and metastatic disease, and inversely associated with poor tumor differentiation [43,44], our series demonstrated that other than a marginal association between tumor LINE-1 hypomethylation and advanced-stage disease (stages III and IV), tumor LINE-1 hypomethylation was not associated with any other clinicopathological features. Tumor LINE-1 hypomethylation associated with advanced-stage disease had been published in several studies but was not conclusive [44–47]. A study designed by Benard et al. demonstrated that LINE-1 methylation of normal tissues was approximately 90%, and 14.2% higher than those of tumor tissues on average [44]. As tumor node metastasis (TNM) stage increased, LINE-1 methylation decreased from 80% (stage I) to 65% (stage III) [44]. Sunami et al. suggested that genomic methylation level might decrease during CRC carcinogenesis and progression, because their data provided evidence of a linear correlation between tumor LINE-1 hypomethylation progression and TNM stage progression [45]. In contrast, Murata et al. [46] showed LINE-1 methylation levels of liver metastases were similar to those of primary tumors (69 ± 11.3%). In addition, a large population-based CRC study [47] including 869 cases demonstrated that LINE-1 methylation levels in tumors were not associated with tumor stage (stages I–IV). Our results showed LINE-1 hypomethylation affecting CRC patients’ outcomes in univariate analysis but not in multivariate analysis. A possible explanation is that TNM impacted patients’ outcomes in univariate and multivariate analysis and our LINE-1 hypomethylation was marginally associated with TNM stage.

Genome-wide-DNA hypomethylation has been associated with genomic and chromosomal instability (CIN) [48–50]. Further, LINE-1 hypomethylation was found to be associated with p53 mutation [13] and activation of proto-oncogenes including MET [51], but inversely correlated with MSI [42,52]. However, our series did not find any association between LINE-1 hypomethylation, mutations of 12 genes studied, and MSI-high. EMAST is a phenomenon of tetranucleotide instability. Our series did not find any association between LINE-1 methylation and EMAST. Until now, there have been no reports mentioning LINE-1 methylation and EMAST.

Although this study collected several types of molecular alterations and had LINE-1 data of normal subjects, its limitation was a sample size inadequate to achieve statistical significance. With the development of next-generation sequencing (NGS), this type of study (especially mutational analysis) should take advantage of NGS to detect the whole length of targeted genes.

## Conclusion

Our study provided evidence that first a mean methylation level of 80% was found in normal subjects, and that this could indicate a potential threshold for pathologic activity. Second, CRC patients’ clinicopathological features, DFS, and OS had no association with tissue LINE-1 hypomethylation in multivariate analysis. Only TNM staging was significantly associated with patients’ outcome. Tumor LINE-1 hypomethylation was marginally associated with advanced stages of CRC. The molecular alterations including specific gene mutations, MSI and EMAST LINE-1 were not associated with tumor LINE-1 hypomethylation.

## Supporting information

S1 Table Molecular alterations of hypo and normal methylation of Line-1 and abbreviations. (XLSX)

S1 Dataset The detailed information of 143 CRC patients including clinicopathological features and molecular alterations. (XLSX)
